# Individualization of risk factors for postoperative complication after lung cancer surgery: a retrospective study

**DOI:** 10.1186/s12893-021-01305-0

**Published:** 2021-07-14

**Authors:** Nozomu Motono, Masahito Ishikawa, Shun Iwai, Yoshihito Iijima, Katsuo Usuda, Hidetaka Uramoto

**Affiliations:** Department of Thoracic Surgery, Kanazawa Medical University, 1-1 Daigaku, Uchinada, Ishikawa 920-0293 Japan

**Keywords:** Postoperative complication, Risk factor, Non-small cell lung cancer, Surgery

## Abstract

**Background:**

The risk factors for postoperative complications after pulmonary resection in patients with non-small cell lung cancer (NSCLC) have not been elucidated.

**Methods:**

Clinical data of 956 patients with NSCLC were analyzed. Patient factors such as sex, age, comorbidities, smoking history, respiratory function, and the lobe involved in lung cancer and operative factors such as operative approach and operative procedures were collected and analyzed.

**Results:**

Male sex (odds ratio [OR]: 1.73, 95% confidence interval [CI]: 1.09–2.75, p = 0.01), coexistence of asthma (OR 2.68, 95% CI 1.19–6.02, p = 0.01), low percentage of forced expiratory volume in 1 s (FEV_1_) (OR 1.41, 95% CI 1.02–1.95, p = 0.03), and lobectomy or greater resection (OR 2.47, 95% CI 1.66–3.68, p < 0.01) were identified as significant risk factors for postoperative complications. Male sex (OR 1.98; 95% CI 1.03–3.81, p = 0.03) and complete video-assisted thoracic surgery and robot-assisted thoracic surgery (OR 1.64; 95% CI 1.09–2.45; p = 0.01) were identified as significant risk factors for postoperative air leakage. Coexistence of asthma (OR 9.97; 95% CI 3.66–27.38; p < 0.01) was identified as a significant risk factor for postoperative atelectasis or pneumonia. Lobectomy or greater resection (OR 19.71; 95% CI 2.70–143.57; p < 0.01) was identified as a significant risk factor for postoperative arrhythmia.

**Conclusion:**

Male sex, coexistence of asthma, low percentage of FEV_1_, and operative procedure were significant risk factors for postoperative complications. Furthermore, risk factors varied according to postoperative complications.

## Introduction

Lung cancer is the leading cause of cancer-related mortality worldwide [1]. The incidence of postoperative complications associated with pulmonary resection for non-small cell lung cancer (NSCLC) was reported to be 9–37% [2–4]. Furthermore, the incidence of postoperative complications associated with lobectomy was 10–50% and it was higher in the elderly [5]. Several postoperative complications might occur after pulmonary resection. Air leakage, pneumonia, atelectasis, and arrhythmia are considered common complications. The incidence of postoperative pulmonary complications after pulmonary resection was reported to be 6–30%. Age, smoking history, and chronic obstructive pulmonary disease are considered significant risk factors for postoperative pulmonary complications [6–10].

Video-assisted thoracic surgery (VATS) for patients with NSCLC has been widely adopted and various studies have reported the advantages of the VATS approach [11–14]. These reports have shown that VATS is associated with less pain, shorter hospital stay, less reduction in the inflammatory immune response, and maintenance of postoperative respiratory function when compared with thoracotomy. However, the relationship between postoperative complications and operative approaches such as VATS in NSCLC patients who have undergone pulmonary resection has not been elucidated.

In the present study, we retrospectively evaluated the risk factors for postoperative complication after pulmonary resection in NCSLC patients, and individualized the risk factors each postoperative complication.

## Materials and methods

### Patients

**Fig. 1:**
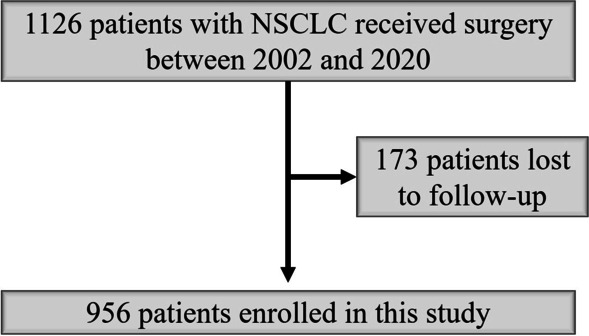
Flowchart of this study

1129 NSCLC patients who underwent pulmonary resection at Kanazawa Medical University between January 2002 and March 2020 were identified. Among these, 173 patients were lost to follow-up, and then, 956 patients were enrolled in the present retrospective study (Fig. 1).

Data including clinical factors such as gender, age, comorbidities, smoking history, respiratory function, and the lobe involved in lung cancer were collected. The average life expectancy of Japanese people is increasing, and since 75 years old or older is defined as the late-stage elderly, it is divided by 75 years old. The comorbidity was evaluated by the Charlson comorbidity index [15]. Furthermore, comorbidities were divided into five categories: interstitial lung disease, malignant disease, asthma, arrhythmia, and angina pectoris. Smoking history was assessed using the Brinkman index, which is calculated by multiplying the number of cigarettes smoked per day by the number of years the subject has been smoking [16]. Respiratory function parameters such as percent-predicted vital capacity (%VC) and forced expiratory volume in 1 s as a percentage of forced vital capacity (FEV_1_%) were collected. In Japan, FEV1.0 of less than 70% is defined as obstructive ventilatory impairment.

### Operative factors

The operative approach was divided into four categories: complete VATS (C-VATS, surgery performed only to provide a monitoring view, wound length less than 8 cm), hybrid VATS (H-VATS, surgery combined with direct vision without rib spreading, wound length more than 8 cm), robot-assisted thoracic surgery (RATS), and thoracotomy. The operative procedure was divided into eight categories: wedge resection, segmentectomy, lobectomy, sleeve lobectomy, lobectomy combined with segmentectomy, lobectomy combined with chest wall resection, bi-lobectomy, and pneumonectomy.

### Postoperative complications

Postoperative complications were categorized into five grades according to the Clavien-Dindo classification system [17]. The Clavien-Dindo classification was established in 1992. It is a simple and feasible grading system for all types of postoperative complications [18]. In 2004, it was modified to allow for the grading of life-threatening complications and long-term disability caused by a complication [19]. This revised version has defined five grades of severity with subgrades (grade I, II, IIIa, IIIb, IVa, IVb, and V) and the suffix “d” (for “disability”) is used to denote any postoperative impairment. This modified version of the Clavien-Dindo classification has been widely used in clinical practice. Major postoperative complications were defined as follow; air leakage which categorized grade IIIa or more, arrhythmia which categorized grade II or more, atelectasis which categorized grade IIIa or more, pneumonia which categorized grade II or more, chylothorax which categorized grade IIIa or more, cerebral infarction which categorized grade II or more, and bronchopleural fistula which categorized grade IIIa or more. Minor but serious postoperative complications were defined as the postoperative complications which categorized grade IIIb or more except for the major postoperative complications. We also divided the postoperative complications into three categories for multivariate analysis of risk factors: air leakage, atelectasis or pneumonia, and arrhythmia.

### Statistical analyses

Pearson’s chi-squared test of independence was used to compare the frequencies of variables. The risk factors related to postoperative complications were analyzed using logistic regression analysis. All statistical analyses were two-sided and statistical significance was set at p < 0.05. Statistical analyses were conducted using the JMP software program version 13.2 (SAS Institute Inc., Cary, NC, USA).

The present study was conducted in accordance with the principles of the Declaration of Helsinki. The Institutional Review Boards of Kanazawa Medical University approved the protocol (approval number: I392) and written informed consent was obtained from all patients.

## Results

### Patient characteristics

The clinical characteristics of the 956 patients are listed in Table 1. Among these, 585 were men and the median age was 69.7 years. The median Brinkman index was 540. Altogether, 540 patients had comorbidities including 135 patients with hypertension, 137 with diabetes mellitus, 22 with interstitial lung disease, 161 with malignant disease (19 with colon cancer, 8 with rectal cancer, 50 with gastric cancer, 16 with breast cancer, 17 with prostate cancer, 12 with bladder cancer, 4 with gallbladder cancer, 14 with thyroid cancer, 4 with renal cancer, 5 with laryngeal cancer, 10 with pharyngeal cancer, and 3 with tongue cancer), 28 with asthma, 26 with arrhythmia (23 with atrial fibrillation, 2 with paroxysmal supraventricular tachycardia, and 1 with atrioventricular block), and 70 with angina pectoris. The median %VC was 100.1% and the median FEV_1_% was 73.7%. The pulmonary lobes resected for NSCLC included right upper lobe in 289 patients, right middle lobe in 58, right lower lobe in 231, left upper lobe in 220, and left lower lobe in 158 patients.

**Table 1:** Patient characteristics

Gender (male/female)	585/371
Age, median (range)	69.7 (22—92)
Comorbidity	550 (57.5%)
Interstitial lung disease (%)	22 (2.3%)
Malignancy (%)	161 (16.8%)
Diabetes mellitus (%)	136 (14.2%)
Asthma (%)	28 (2.9%)
Arrhythmia (%)	26 (2.7%)
Angina pectoris (%)	70 (7.3%)
Charlson comorbidity index (0/1/2/3/4/5/6/7)	539/201/152/47/13/3/1/1
Smoking index, median (range)	540 (0–3600)
%VC, median (range)	100.1 (51.5–177.7)
FEV_1_%, median (range)	73.7 (30.5–99.4)
Lobe of lung cancer	
RU/RM/RL/LU/LL	289/58/231/220/158
Operative approach	
RATS/C-VATS/H-VATS/Open	12/329/509/106
Operative procedure	
Part/Seg/Lob/Sleeve Lob/Lob + Seg/Lob + CW/Bilob/Pneumo	173/74/643/6/3/12/18/27
Stage (IA/IB/IIA/IIB/IIIA/IIIB/IV/yIA/yIIA)	563/167/60/81/70/5/4/5/1
Histology (Ad/Sq/LCNEC/AdSq/Pleo/Large/Carci)	726/180/19/11/9/4/7
Postoperative complication	257 (26.8%)
Clavien-Dindo grade (0/1/2/3a/3b)	699/1/101/146/9
Air leakage (%)	112 (11.7%)
Arrhythmia (%)	58 (6.1%)
Atelectasis (%)	26 (2.7%)
Pneumonia (%)	21 (2.2%)
Chylothorax	6 (0.6%)
Cerebral infarction (%)	5 (0.5%)
Bronchopleural fistula	3 (0.3%)
30-day mortality (%)	1 (0.1%)
90-day mortality (%)	3 (0.3%)
Postoperative hospital-stay, days, median (range)	12 (3–100)

*%VC* vital capacity, *FEV1%* forced expiratory volume % in one second, *RU* right upper, *RM* right middle, *RL* right lower, *LU* left upper, *LL* left lower, *RATS* Robot-assisted Thoracic Surgery, *C* complete, *VATS* video-assisted thoracic surgery, *H* hybrid, *Part* partial resection, *Seg* segmentectomy, *Lob* lobectomy, *CW* chest wall resection, *Bilob* bilobectomy, *Pneumo* pneumonectomy, *p* pathological, *y* yield to treatment, *Ad* adenocarcinoma, *Sq* squamous cell carcinoma, *LCNEC* large cell neuroendocrine carcinoma, *AdSq* adenosquamous cell carcinoma, *Pleo* pleomorphic carcinoma, *Large* large cell carcinoma, *Carci* carcinoid

### Operative factors

C-VATS was performed in 329 patients, H-VATS in 509 patients, RATS in 12 patients, and thoracotomy in 106 patients. Wedge resection was performed in 173 patients, segmentectomy in 74, lobectomy in 643, sleeve lobectomy in 6, lobectomy combined with segmentectomy in 3, lobectomy combined with chest wall resection in 12, bilobectomy in 18, and pneumonectomy in 27 patients.

### Postoperative complications

Postoperative complications were observed in 257 patients (26.8%). Clavien-Dindo grade I complications were noted in 1 patient, grade II in 101, grade IIIa in 146, and grade IIIb in 9 patients. Major postoperative complications included air leakage in 122 patients (Clavian-Dindo grade II in 6, IIIa in 105 and IIIb in 1), arrhythmia in 58 (atrial fibrillation in 55 patients, paroxysmal supraventricular tachycardia in 2 patients, and ventricular tachycardia in 1 patient, grade II in 56, IIIa in 2), atelectasis in 26 (grade II in 1 and IIIa in 25), pneumonia in 21 (grade II in 20 and IIIa in 1), chylothorax in 6, cerebral infarction in 5, and bronchopleural fistula in 3 patients. Minor but serious postoperative complications included postoperative bleeding in 3 patients, right middle lobe torsion in 1 patient, and right middle lobe congestion in 1 patient. All these complications were resolved by surgery. Although postoperative death was observed in 1 case, the cause was not resolved due to sudden death after discharge. The median duration of postoperative hospital stay was 12 days.

### Univariate analysis

The relationship between patient characteristics or operative factors and postoperative complications was analyzed (Table 2). Postoperative complications were more likely to be associated with male sex (p < 0.01), smoking history (p < 0.01), coexistence of asthma (p = 0.01), low FEV_1_% (p < 0.01), and lobectomy or greater resection (p < 0.02). The results of the relationship between patient characteristics or operative factors and postoperative air leakage are presented in Table 3. Postoperative air leakage was more likely to be associated with male sex (p < 0.01), smoking history (p < 0.01), coexistence of asthma (p < 0.01), low FEV_1_% (p < 0.01), and C-VATS and RATS (p = 0.02). The relationship between patient characteristics or operative factors and postoperative atelectasis or pneumonia was analyzed and the results are presented in Table 4. Postoperative atelectasis or pneumonia was more likely to be associated with male sex (p = 0.01), smoking history (p < 0.01), coexistence of asthma (p < 0.01), and low FEV_1_% (p = 0.03). The relationship between patient characteristics or operative factors and postoperative arrhythmia was analyzed and the results are presented in Table 5. Postoperative arrhythmia was more likely to be associated with H-VATS or thoracotomy (p = 0.02) and with lobectomy or greater resection (p < 0.01). Significant risk factors for other postoperative complications could not be assessed due to small number of patients with these complications.

**Table 2:** Relationship between patient characteristics or operative factors and postoperative complication

	Postoperative complications		p value
	Present	Absent	
Gender			
Male	193 (33%)	392 (67%)	< 0.01
Female	64 (17%)	307 (83%)	
Age			
< 75 years	183 (26%)	516 (74%)	0.41
≥ 75 years	74 (29%)	183 (71%)	
Smoking history			
Present	196 (33%)	399 (67%)	< 0.01
Absent	61 (17%)	300 (83%)	
Charlson Comorbidity index			
≧3	13 (20%)	52 (80%)	0.19
< 3	245 (27%)	647 (73%)	
Coexistence of Interstitial lung disease			
Present	4 (18%)	18 (82%)	0.35
Absent	253 (27%)	681 (73%)	
Coexistence of diabetes mellitus			
Present	39 (29%)	97 (71%)	0.61
Absent	218 (27%)	602 (73%)	
Coexistence of malignancy			
Present	36 (22%)	125 (78%)	0.15
Absent	221(28%)	704 (72%)	
Coexistence of asthma			
Present	13 (46%)	15 (54%)	0.01
Absent	244 (26%)	684 (73%)	
Coexistence of arrhythmia or angina pectoris			
Present	32 (32%)	66 (68%)	0.17
Absent	225 (26%)	633 (74%)	
FEV_1_%			
< 70%	115 (35%)	218 (65%)	< 0.01
≥ 70%	142 (23%)	481 (77%)	
Lobe			
RU	84 (29%)	205 (71%)	0.07
RM	11 (19%)	47 (81%)	
RL	63 (27%)	168 (73%)	
LU	72 (32%)	148 (68%)	
LL	27 (17%)	131 (83%)	
Operative approach			
C-VATS + RATS	88 (26%)	253 (74%)	0.57
H-VATS + Open	169 (27%)	446 (73%)	
Operative procedure			
Lobectomy or more	219 (31%)	490(69%)	< 0.01
Sublobar resection	38 (15%)	209 (85%)	

*FEV1%* forced expiratory volume % in one second, *RU* right upper, *RM* right middle, *RL* right lower, *LU* left upper, *LL* left lower, *RATS* Robot-assisted Thoracic Surgery, *C* complete, *VATS* video-assisted thoracic surgery, *H* hybrid

**Table 3:** Relationship between patient characteristics or operative factors and postoperative air leakage

	Air leakage		p value
	Present	Absent	
Gender			
Male	88 (15%)	497 (85%)	< 0.01
Female	24 (7%)	347 (94%)	
Age			
< 75 y	77 (11%)	622 (89%)	0.26
≥ 75 y	35 (14%)	222 (86%)	
Smoking history			
Present	87 (15%)	508 (85%)	< 0.01
Absent	25 (7%)	336 (94%)	
Coexistence of interstitial lung disease			
Present	0 (0%)	22 (100%)	0.08
Absent	112 (12%)	822 (88%)	
Coexistence of diabetes mellitus			
Present	18 (13%)	118 (87%)	0.55
Absent	94 (11%)	726 (89%)	
Coexistence of malignancy			
Present	21 (13%)	140 (87%)	0.56
Absent	91 (12%)	704 (88%)	
Coexistence of asthma			
Present	3 (10%)	25 (90%)	0.86
Absent	109 (12%)	819 (88%)	
Coexistence of arrhythmia or angina pectoris			
Present	11 (11%)	87 (89%)	0.87
Absent	101 (12%)	757 (88%)	
FEV_1_%			
< 70%	52 (16%)	281 (84%)	< 0.01
≥ 70%	60 (10%)	563 (90%)	
Lobe			
RU	33 (11%)	256 (89%)	0.18
RM	4 (7%)	54 (93%)	
RL	28 (12%)	203 (88%)	
LU	34 (15%)	186 (85%)	
LL	13 (8%)	145 (92%)	
Operative approach			
C-VATS + RATS	51 (15%)	290 (85%)	0.02
H-VATS + Open	61 (10%)	554 (90%)	
Operative procedure			
Lobectomy or more	87 (12%)	622 (88%)	0.36
Sublobar resection	25 (10%)	222 (90%)	

*FEV1%* forced expiratory volume % in one second, *RU* right upper, *RM* right middle, *RL* right lower, *LU* left upper, *LL* left lower, *C* complete, *VATS* video-assisted thoracic surgery, *RATS* Robot-assisted Thoracic Surgery, *H* hybrid

**Table 4:** Relationship between patient characteristics or operative factors and postoperative atelectasis or pneumonia

	Atelectasis or pneumonia		p value
	Present	Absent	
Gender			
Male	37 (6%)	548 (94%)	0.01
Female	10 (3%)	361 (97%)	
Age			
< 75 years	31 (4%)	668 (96%)	0.25
≥ 75 years	16 (6%)	241 (94%)	
Smoking history			
Present	39 (6%)	556 (94%)	< 0.01
Absent	8 (2%)	353 (98%)	
Coexistence of ILD			
Present	2 (9%)	20 (91%)	0.35
Absent	45 (5%)	889 (95%)	
Coexistence of diabetes mellitus			
Present	6 (4%)	130 (96%)	0.76
Absent	41 (5%)	779 (95%)	
Coexistence of malignancy			
Present	8 (5%)	153 (95%)	0.97
Absent	39 (5%)	756 (95%)	
Coexistence of asthma			
Present	7 (25%)	21 (75%)	< 0.01
Absent	40 (4%)	888 (96%)	
FEV_1_%			
< 70%	23 (7%)	310 (93%)	0.03
≥ 70%	24 (4%)	599 (96%)	
Coexistence of arrhythmia or angina pectoris			
Present	8 (8%)	90 (92%)	0.11
Absent	39 (5%)	819 (95%)	
Lobe			
RU	18 (6%)	271 (94%)	0.23
RM	2 (3%)	56 (97%)	
RL	10 (4%)	221 (96%)	
LU	14 (6%)	206 (94%)	
LL	3 (2%)	155 (98%)	
Operative approach			
C-VATS + RATS	12 (4%)	329 (96%)	0.13
H-VATS + Open	35 (6%)	580 (94%)	
Operative procedure			
Lobectomy or more	38 (5%)	671 (95%)	0.28
Sublobar resection	9 (4%)	238 (96%)	

*FEV1%* forced expiratory volume % in one second, *RU* right upper, *RM* right middle, *RL* right lower, *LU* left upper, *LL* left lower, *C* complete, *VATS* video-assisted thoracic surgery, *RATS* Robot-assisted Thoracic Surgery, *H* hybrid

**Table 5:** Relationship between patient characteristics or operative factors and postoperative arrhythmia

	Arrhythmia		p value
	Present	Absent	
Gender			
Male	38 (7%)	547 (93%)	0.48
Female	20 (5%)	351 (95%)	
Age			
< 75 years	43 (6%)	656 (94%)	0.85
≥ 75 years	15 (6%)	242 (94%)	
Smoking history			
Present	40 (7%)	555 (93%)	0.27
Absent	18 (5%)	343 (95%)	
Coexistence of interstitial lung disease			
Present	2 (9%)	20 (91%)	0.54
Absent	56 (6%)	878 (94%)	
Coexistence of diabetes mellitus			
Present	5 (4%)	131 (96%)	0.21
Absent	53 (7%)	767 (93%)	
Coexistence of malignancy			
Present	5 (3%)	156 (97%)	0.08
Absent	53 (7%)	742 (93%)	
Coexistence of asthma			
Present	1 (4%)	27 (96%)	0.57
Absent	57 (6%)	871 (94%)	
Coexistence of arrhythmia or angina pectoris			
Present	8 (8%)	90 (92%)	0.35
Absent	50 (6%)	808 (94%)	
FEV_1_%			
< 70%	26 (8%)	307 (92%)	0.09
≥ 70%	32 (5%)	591 (95%)	
Lobe			
RU	21 (7%)	268 (93%)	0.30
RM	3 (5%)	55 (95%)	
RL	14 (6%)	217 (94%)	
LU	16 (7%)	204 (93%)	
LL	4 (2%)	154 (98%)	
Operative approach			
C-VATS + RATS	13 (4%)	328 (96%)	0.02
H-VATS + Open	45 (7%)	570 (93%)	
Operative procedure			
Lobectomy or more	57 (8%)	652 (92%)	< 0.01
Sublobar resection	1 (0.4%)	246 (99.6%)	

*FEV1%* forced expiratory volume % in one second, *RU* right upper, *RM* right middle, *RL* right lower, *LU* left upper, *LL* left lower, *C* complete, *VATS* video-assisted thoracic surgery, *RATS* Robot-assisted Thoracic Surgery, *H* hybrid

### Multivariate analysis

The multivariate analyses of risk factors for postoperative complications are presented in Table 6. Male sex (odds ratio [OR]: 1.73, 95% confidence interval [CI] 1.09–2.75, p = 0.01), coexistence of asthma (OR 2.68, 95% CI 1.19–6.02, p = 0.01), low FEV_1_% (OR 1.41, 95% CI 1.02–1.95; p = 0.03), and lobectomy or greater resection (OR 2.47, 95% CI 1.66–3.68, p < 0.01) were identified as significant risk factors for postoperative complications. Results of the multivariate analyses of risk factors for postoperative air leakage, atelectasis, pneumonia, and arrhythmia are presented in Table 7. Male sex (OR 1.98, 95% CI 1.03–3.81, p = 0.03) and C-VATS and RATS (OR 1.64, 95% CI 1.09–2.45, p = 0.01) were identified as significant risk factors for postoperative air leakage. Coexistence of asthma (OR 9.97, 95% CI 3.66–27.38, p < 0.01) was identified as a significant risk factor for postoperative atelectasis or pneumonia. Lobectomy or greater resection (OR 19.71, 95% CI 2.70–143.57, p < 0.01) was identified as a significant risk factor for postoperative arrhythmia.

**Table 6:** Multivariate analysis of risk factors for postoperative complications

Risk factors	*p*	OR	95% CI
Gender			
Male	0.01	1.73	1.09–2.75
Smoking history			
Present	0.08	1.50	0.94–2.40
Asthma			
Present	0.01	2.68	1.19–6.02
FEV_1_%			
< 70%	0.03	1.41	1.02–1.95
Operative procedure			
Lobectomy or more	< 0.01	2.47	1.66–3.68

*O.R.* odds ratio, *CI* confidence interval, *FEV1%* forced expiratory volume % in one second

**Table 7:** Multivariate analysis of risk factors for postoperative complications

Postoperative complications	Risk factors		*p*	O.R	95% CI
Air leakage	Gender	Male	0.03	1.98	1.03–3.81
	Smoking history	Present	0.45	1.28	0.66–2.47
	FEV_1_%	< 70%	0.15	1.35	0.88–2.07
	Operative approach	C-VATS + RATS	0.01	1.64	1.09–2.45
Atelectasis or pneumonia	Gender	Male	0.21	1.85	0.69–4.99
	Smoking history	Present	0.14	2.14	0.77–5.95
	Asthma	Present	< 0.01	9.97	3.66–27.38
	FEV_1_%	< 70%	0.65	1.15	0.61–2.16
Arrhythmia	Operative approach	C-VATS + RATS	0.15	0.63	0.33–1.19
	Operative procedure	Lobectomy or more	< 0.01	19.71	2.70–143.57

*O.R.* odds ratio, *CI* confidence interval, *FEV1%* forced expiratory volume % in one second, *C* complete, *VATS* video-assisted thoracic surgery, *RATS* Robot-assisted Thoracic Surgery

## Discussion

In the present study, we analyzed the risk factors for postoperative complications in patients who underwent pulmonary resection for NSCLC. Several factors such as age, smoking history, Charlson comorbidity index, operative approach, and the type of operative procedure have been reported as risk factors for postoperative complications in NSCLC patients who had undergone pulmonary resection [5, 20, 21]. In the present study, although Charlson comorbidity index was not significant risk factor for postoperative complications, sex, coexistence of asthma, low FEV_1_%, and the type of operative procedure were significant risk factors for postoperative complications. Furthermore, although the VATS approach has been reported to be less invasive than thoracotomy [11–14], it has not been clarified whether it reduces postoperative complications in the present study.

Postoperative air leakage is the most common complication after pulmonary resection, with a reported incident rate of 15–18% [22]. Although patient factors such as emphysematous lung or large parenchymal resection are considered to increase the risk of air leakage [23], male sex and C-VATS or RATS approach were significant risk factors for postoperative air leakage in the present study. Although the VATS or the RATS approach could provide a good field of view through the camera monitor, it is possible that air leakage was missed due to improper use of these systems in the initial days of their use.

Postoperative pulmonary complications are also common after pulmonary resection. And previous studies have reported an incidence rate of 6–29% [6–9, 20, 23, 24]. In these reports, age, smoking history, chronic obstructive pulmonary disease, and thoracotomy have been reported as risk factors for postoperative pulmonary complications. In the present study, coexistence of asthma was a significant risk factor for postoperative atelectasis or pneumonia. Since asthma tends to produce mucus, it can lead to the development of postoperative atelectasis or pneumonia due to mucus plugging. However, male sex, smoking history, and low FEV_1_% were significantly associated with postoperative atelectasis or pneumonia in the univariate analysis. Thus, these factors might also be important risk factors for the development of postoperative pulmonary complications.

Atrial fibrillation is the most common type of postoperative arrhythmia in patients who have undergone pulmonary resection, with a reported incidence rate of 9–19% [25–28]. Although the mechanism of atrial fibrillation after non-cardiac thoracic surgery is unknown, several factors including male sex, age, amount of lung resection, previous episode of congestive heart failure, prior arrhythmia, and neoadjuvant chemotherapy were reported as the risk factors for postoperative arrhythmia [25–28]. In the present study, the incidence rate of postoperative arrhythmia was 6.1% and the type of operative procedure (lobectomy or greater resection) was a significant risk factor for postoperative arrhythmia. It is suggested that the risk of postoperative arrhythmia might be related to the amount of lung resection.

Reportedly, VATS is associated with less pain, less reduction in the inflammatory immune response, and maintenance of postoperative respiratory function when compared with thoracotomy. Therefore, VATS is considered a less invasive procedure [11–14]. On the other hand, it was reported that VATS dose not reduce the incidence rate of postoperative atrial fibrillation after pulmonary lobectomy [29]. It was suggested that autonomic denervation and stress-mediated neurohumoral mechanisms resulting from anatomic pulmonary resection, and not an incision-related effect. In the univariate analysis, the incidence rate of postoperative arrhythmia was significantly higher in patients who underwent H-VATS or thoracotomy. However, the operative approach was not a significant risk factor in the multivariate analysis in the present study. Although C-VATS or RATS are less invasive, the operative approach might not reduce the incidence rate of postoperative arrhythmia.

The present study has several limitations. The study had a retrospective design and there was a possibility of unobserved cofounding and selection bias. Another limitation is that the present study was performed at a single institution.

## Conclusions

Our findings analyzed the risk factors for postoperative complications in patients who underwent pulmonary resection for NSCLC. Male sex, coexistence of asthma, low FEV_1_%, and the type of operative procedure were significant risk factors for postoperative complications. Risk factors varied according to postoperative complications. Male sex and C-VATS or RATS approach were significant risk factors for postoperative air leakage. Coexistence of asthma was a significant risk factor for postoperative atelectasis or pneumonia. The type of operative procedure (lobectomy or greater resection) was a significant risk factor for postoperative arrhythmia. Because there is no specific risk score for the occurrence of postoperative complications after pulmonary resection, and further studies are needed. In addition, further analysis of risk factors involved in the development of individual complications after pulmonary resection is considered to be necessary.

## Data Availability

The datasets generated and/or analyzed during the current study are not publicly available due to [our institutional restrictions e.g. them containing information that could compromise research participant privacy/consent], but are available from the corresponding author on reasonable request.

## Abbreviations

NSCLC: Non-small cell lung cancer
VATS: Video-assisted thoracic surgery
%VC: Vital capacity
FEV1%: Forced expiratory volume % in one second, robot-assisted thoracic surgery
